# Quorum Sensing Controls Flagellar Morphogenesis in *Burkholderia glumae*

**DOI:** 10.1371/journal.pone.0084831

**Published:** 2014-01-08

**Authors:** Moon Sun Jang, Eunhye Goo, Jae Hyung An, Jinwoo Kim, Ingyu Hwang

**Affiliations:** 1 Department of Agricultural Biotechnology, Seoul National University, Seoul, Republic of Korea; 2 Division of Applied Life Science and Institute of Agriculture and Life Sciences, Gyeongsang National University, Jinju, Republic of Korea; Indian Institute of Science, India

## Abstract

*Burkholderia glumae* is a motile plant pathogenic bacterium that has multiple polar flagella and one LuxR/LuxI-type quorum sensing (QS) system, TofR/TofI. A QS-dependent transcriptional regulator, QsmR, activates flagellar master regulator *flhDC* genes. FlhDC subsequently activates flagellar gene expression in *B*. *glumae* at 37°C. Here, we confirm that the interplay between QS and temperature is critical for normal polar flagellar morphogenesis in *B*. *glumae*. In the wild-type bacterium, flagellar gene expression and flagellar number were greater at 28°C compared to 37°C. The QS-dependent *flhC* gene was significantly expressed at 28°C in two QS-defective (*tofI*::Ω and *qsmR*::Ω) mutants. Thus, flagella were present in both *tofI*::Ω and *qsmR*::Ω mutants at 28°C, but were absent at 37°C. Most *tofI*::Ω and *qsmR*::Ω mutant cells possessed polar or nonpolar flagella at 28°C. Nonpolarly flagellated cells processing flagella around cell surface of both *tofI*::Ω and *qsmR*::Ω mutants exhibited tumbling and spinning movements. The *flhF* gene encoding GTPase involved in regulating the correct placement of flagella in other bacteria was expressed in QS mutants in a FlhDC-dependent manner at 28°C. However, FlhF was mislocalized in QS mutants, and was associated with nonpolar flagellar formation in QS mutants at 28°C. These results indicate that QS-independent expression of flagellar genes at 28°C allows flagellar biogenesis, but is not sufficient for normal polar flagellar morphogenesis in *B*. *glumae*. Our findings demonstrate that QS functions together with temperature to control flagellar morphogenesis in *B*. *glumae*.

## Introduction

Structural and physiological adaptations of bacteria facilitate their survival under various stressful environmental conditions. Such adaptations often require the coordination of multiple regulatory systems to control the expression of related genes [1]. One of the best studied examples of bacterial responses to environmental changes is the morphological development of flagella in bacteria to allow motility. More than 50 genes, in addition to regulatory proteins and chemosensory machinery, are required for the biogenesis and assembly of bacterial flagella [2]–[4]. Flagellar biosynthesis genes are highly conserved in bacteria, and their expression is regulated by several known, but not universal, master regulators (including FlhDC, CtrA, VisNR, and FleQ) that act in a hierarchical cascade [5], [6].

Three types of flagellar morphology (polar, peritrichous, and lateral) are determined by a combination of bacterial genetic factors and certain environmental conditions [7]–[9]. *Pseudomonas aeruginosa* and *Vibrio cholerae* possess single polar flagella, whereas *Escherichia coli* and *Salmonella enterica* have peritrichous flagella [7], and *Selenomonas ruminantium* has lateral flagella [8]. *Vibrio parahaemolyticus* has two flagellar systems, one for a single polar flagellum and one for lateral flagella, with these systems being activated by different growth conditions [9].

Two nucleotide-binding proteins, GTPase and ATPase, are involved in the correct localization of cellular components [10]. For example, the signal recognition particle (SRP)-like GTPase FlhF is essential for the placement and assembly of flagella in many polar and peritrichous bacteria [11]–[16]. In polarly flagellated bacteria, such as *Vibrio* and *Pseudomonas* species, FlhF and FlhG (a GTPase activating protein) are involved in regulating flagellar number and positioning, with the *flhF* and *flhG* genes being under the control of master regulators [2], [12], [13], [17], [18]. However, details about the molecular mechanisms that determine flagellar number and morphology in bacteria remain poorly understood.

In addition to the effects of certain environmental factors, such as pH and temperature, on bacterial flagellar formation, quorum sensing (QS) is important for flagellar biosynthesis and cell motility in different bacterial species [19]–[25]. QS is a coordinated gene regulatory system that controls the social behaviors of bacterial populations in response to cell density. Such behaviors include virulence, biofilm formation, motility, protein secretion, and toxin production [24]–[29].

In our previous study, we demonstrated that QS positively regulates the expression of flagellar biosynthesis genes in *Burkholderia glumae*, the causal agent of rice panicle blight, at 37°C [24]. QS-dependent flagellar formation is important for virulence in *B*. *glumae* [24]. *B*. *glumae* is motile, has multiple polar flagella, and possesses one LuxR/LuxI-type QS system, TofI/TofR. TofI is responsible for the biosynthesis of *N*-octanoyl-homoserine lactone (C8-HSL), whereas TofR is the cognate receptor for C8-HSL [27]. TofR-C8-HSL regulates various processes in *B*. *glumae*, including toxoflavin production, flagellar biosynthesis, protein secretion, and oxalate biosynthesis [24], [27], [30], [31]. TofR-C8-HSL also activates an IclR-type transcriptional regulator gene, *qsmR*, which, in turn, activates flagellar master regulator *flhDC* genes [24]. FlhDC subsequently activates expression of flagellar biosynthesis genes in *B*. *glumae* [24].

In a previous study, the wild-type *B*. *glumae* strain, BGR1, produced polar flagella; however, flagella and swimming motility were not observed in two QS-defective (*tofI*::Ω and *qsmR*::Ω) mutants at 37°C [24]. This finding indicated that QS positively regulates the expression of flagellar biosynthesis genes in *B*. *glumae* at 37°C [24]. Yet, QS mutants were motile and possessed polar flagella when sampled from the edge of the swimming ring in plates containing soft agar at 28°C [24].

In the present study, we hypothesized that the flagellar morphology in *B. glumae* is influenced by a combination of temperature and QS, based on our previous observation showing that QS mutants are motile at 28°C but not at 37°C [24]. We investigated flagellar morphology and individual cell movement of wild-type *B*. *glumae* and QS mutants sampled from different locations of the swimming region in agar plate assays. By understanding the mechanisms that regulate flagellar formation in *B*. *glumae*, biological control techniques could be developed that hinder this process and, thus, prevent the spread of this highly virulent disease.

## Results

### Flagellar number depends on temperature in the wild-type BGR1

**Figure 1:**
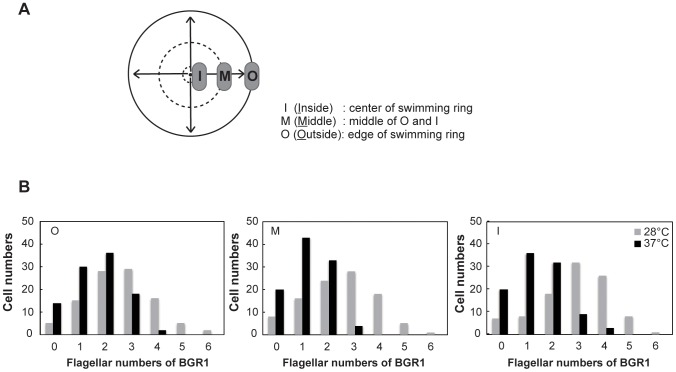
Flagellar number of wild-type strain BGR1 at 28°C and 37°C. **A**. Schematic diagram showing the three divisions of the motility ring on 0.3% soft agar plates. Dotted and solid lines indicate the swimming regions, while the arrows indicate the direction of movement, of motile cells. Outside (O: edge of swimming ring), inside (I: center of swimming ring), and middle (M: middle of O and I) indicate the regions of the swim assay plates from which bacterial samples were taken. **B**. Distribution of BGR1 wild-type cells that had different numbers of flagella that were sampled from the O, M, I regions of the swim assay plates at 28°C and 37°C. Cells were counted based on TEM images (n = 100).

To determine whether temperature is involved in *B. glumae* flagellar formation, the flagellar numbers of individual cells in the wild-type BGR1 were counted on TEM images. At 37°C, more than 77% of the examined flagellated wild-type cells (n = 100 in total) possessed one or two polar flagella in the O, M, and I regions. In contrast, at 28°C, more than 76% of the examined flagellated wild-type cells possessed two to four polar flagella in the O, M, and I regions (Figure 1B).

### Expression of flagellar biosynthesis genes is elevated at 28°C

**Figure 2:**
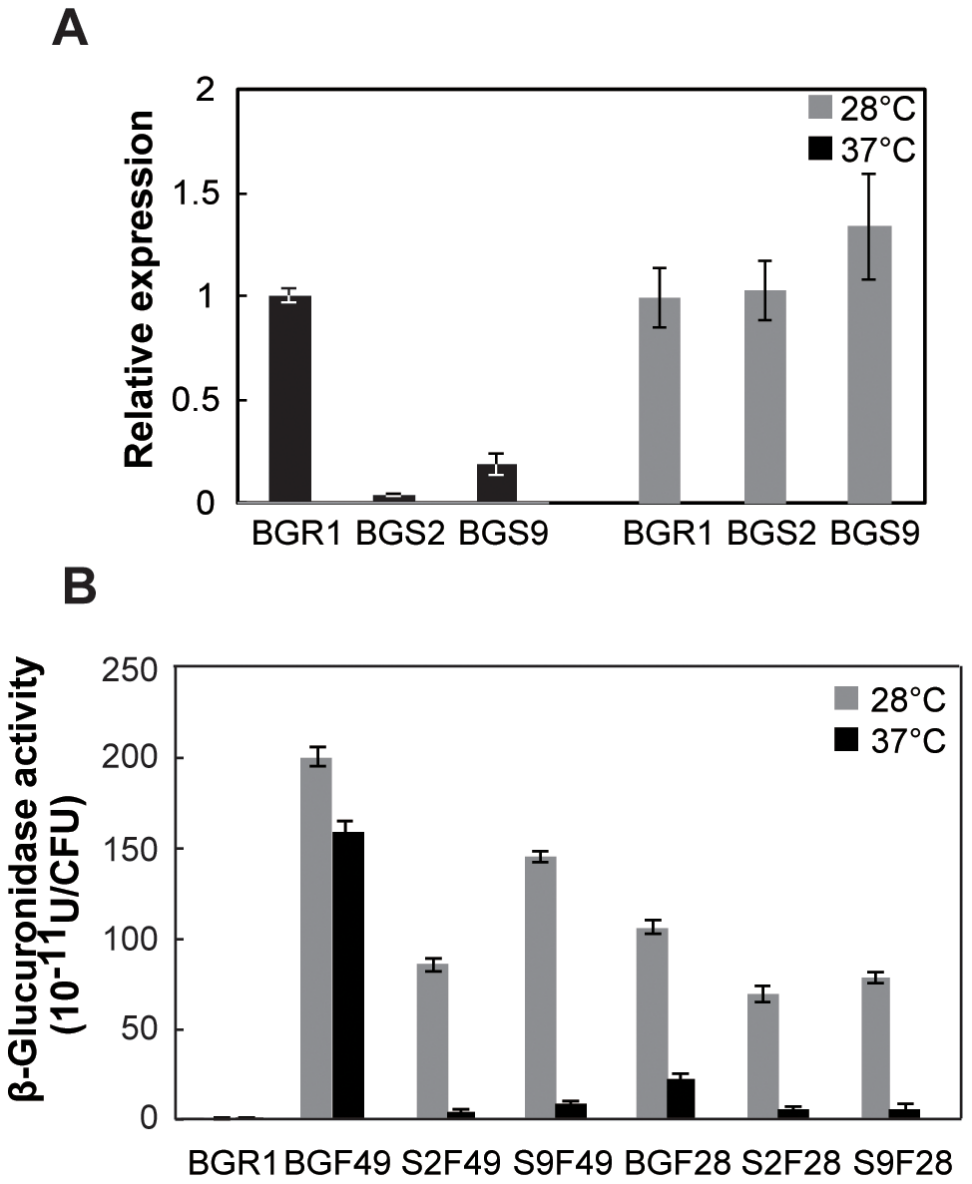
Expression of *flhC* and other flagellar genes in the QS mutants at 28°C and 37°C. **A**. Expression levels of *flhC* in the wild-type BGR1, the *tofI* mutant BGS2, and the *qsmR* mutant BGS9 at 28°C, based on qRT-PCR analysis. Vertical lines indicate the standard deviations of three independent experiments. **B**. Expression levels of *fliC* and *flgK* genes were assessed in the wild-type BGR1, the *tofI* mutant BGS2, and the *qsmR* mutant BGS9 at 28°C and 37°C. BGF49, BGR1 *fliC*::Tn*3*-*gusA49*; S2F49, BGS2 *fliC*::Tn*3*-*gusA49*; S9F49, BGS9 *fliC*::Tn*3*-*gusA49*; BGF28, BGR1 *flgK*::Tn*3*-*gusA28*; S2F28, BGS2 *flgK*::Tn*3*-*gusA28*; S9F28, BGS9 *flgK*::Tn*3*- *gusA28*.

To determine whether the expression of flagellar genes is more elevated at 28°C compared to 37°C, we first measured the expression of the flagellar master regulator *flhC* gene in the wild-type BGR1, the *tofI*::Ω mutant BGS2, and the *qsmR*::Ω mutant BGS9 at 28°C. At 28°C, the *flhC* gene was expressed in the wild-type BGR1, BGS2 mutant, and BGS9 mutant at statistically equivalent levels (Figure 2A). We then predict that other flagellar genes in the BGS2 mutant and the BGS9 mutant might be expressed at 28°C. We examined the expression levels of *fliC* and *flgK* genes in the wild-type BGR1, BGS2 mutant, and BGS9 mutant at both 28°C and 37°C. Higher expression levels were obtained for both *fliC* and *flgK* genes in the wild-type BGR1 at 28°C compared to 37°C (Figure 2B). The QS mutants showed very little *fliC* or *flgK* expression at 37°C, demonstrating that their expression is dependent on QS at 37°C. In contrast, the two genes were expressed at significant levels in the QS mutants at 28°C, but were not as pronounced as in the wild-type BGR1 (Figure 2B). These results were consistent with the observation that *flhC* is expressed in QS mutants at 28°C.

### QS and temperature influence flagellar biogenesis and morphogenesis

**Figure 3:**
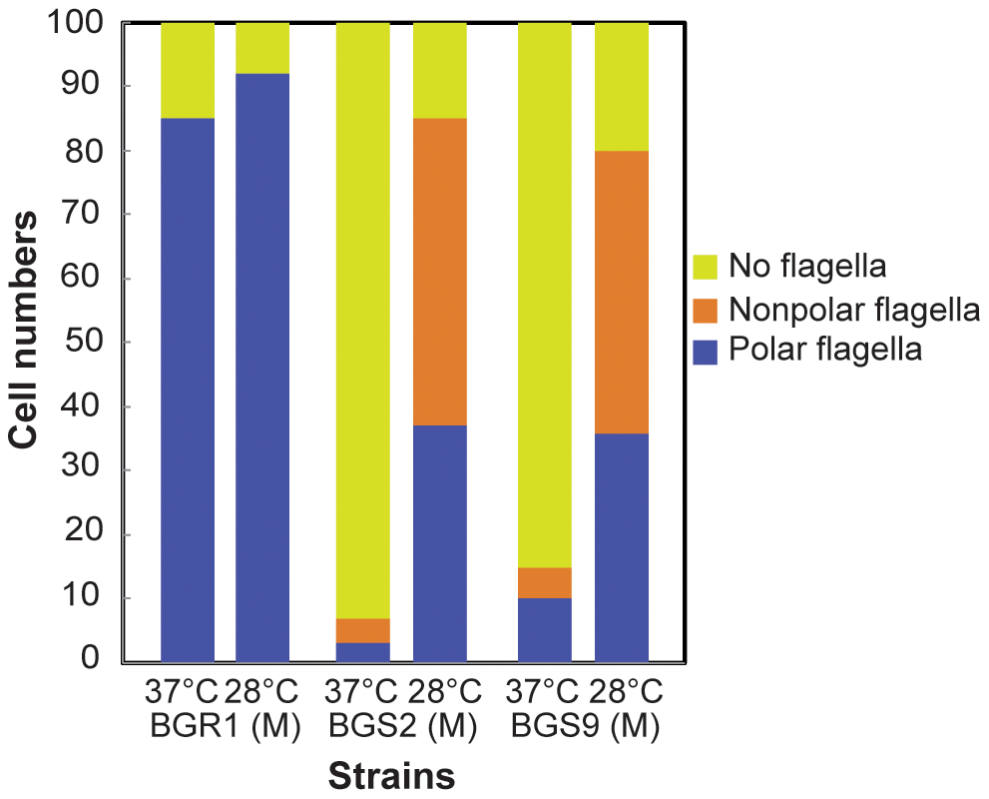
Proportions of 100 individual wild-type BGR1 and the QS mutants processing different flagellar morphology. Bacteria were cultured on swim assay plates for 20°C or 37°C, and samples were collected from the M region of the swim assay plates. Flagellar placement in each strain was determined by analyzing TEM images. Cells with nonpolar flagella possessed flagella around cell surface not at the pole.

**Figure 4:**
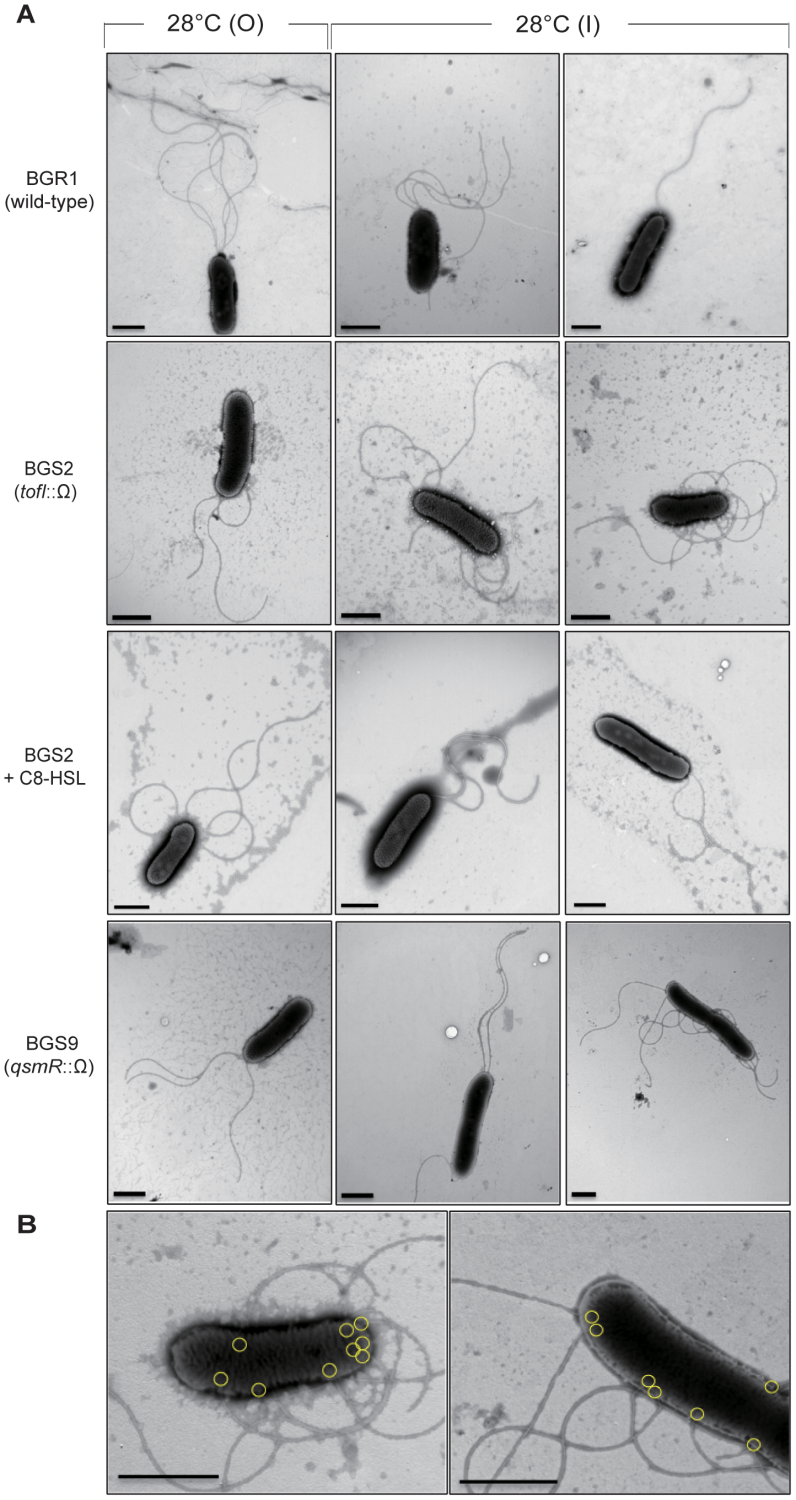
Flagellar morphology of the wild-type BGR1 and the QS mutants. **A**. TEM images showing polar and nonpolar flagellar formation in the wild-type BGR1, the *tofI* mutant BGS2, and the *tofI* mutant BGS2 cells supplemented with 1 µM of C8-HSL, and the *qsmR* mutant BGS9 cells taken from the O and I regions of the swim assay plates after culture for 20 h at 28°C. **B**. Magnified TEM images of nonpolar flagella in the *tofI* mutant BGS2 cells and the *qsmR* mutant BGS9 cells. Yellow circles indicate the sites of flagellar formation. Bars = 1 µm.

We previously reported that the BGS2 mutant and the BGS9 mutant exhibited swimming motility in soft agar at 28°C, but not at 37°C. Therefore, we investigated whether flagellar formation and morphology are influenced by temperature, in addition to QS. At 37°C, most of the examined BGS2 and BGS9 mutant cells (93 and 85 out of 100 cells for each mutant) were aflagellate, as expected (Figure 3). However, at 28°C, 80–85% of the BGS2 and BGS9 mutant cells sampled from the M region of the swim assay plates possessed polar or nonpolar flagella, with approximately half of these cells having nonploar flagella (Figure 3). All of the BGS2 and BGS9 mutant cells sampled from the O region possessed normal polar flagella, as we previously reported (a representative single cell is shown in Figure 4A). Mixtures of cells possessing polar or nonpolar containing bipolar, or peritrichous flagella were observed in the I region (examples are presented in Figures 4A and B). The exogenous addition of 1 µM C8-HSL to the soft agar assay plates led to the recovery of normal polar flagellar morphology in mutant BGS2 cells present in the I region (Figure 4A).

### Lack of QS affects directional swimming movement

**Figure 5:**
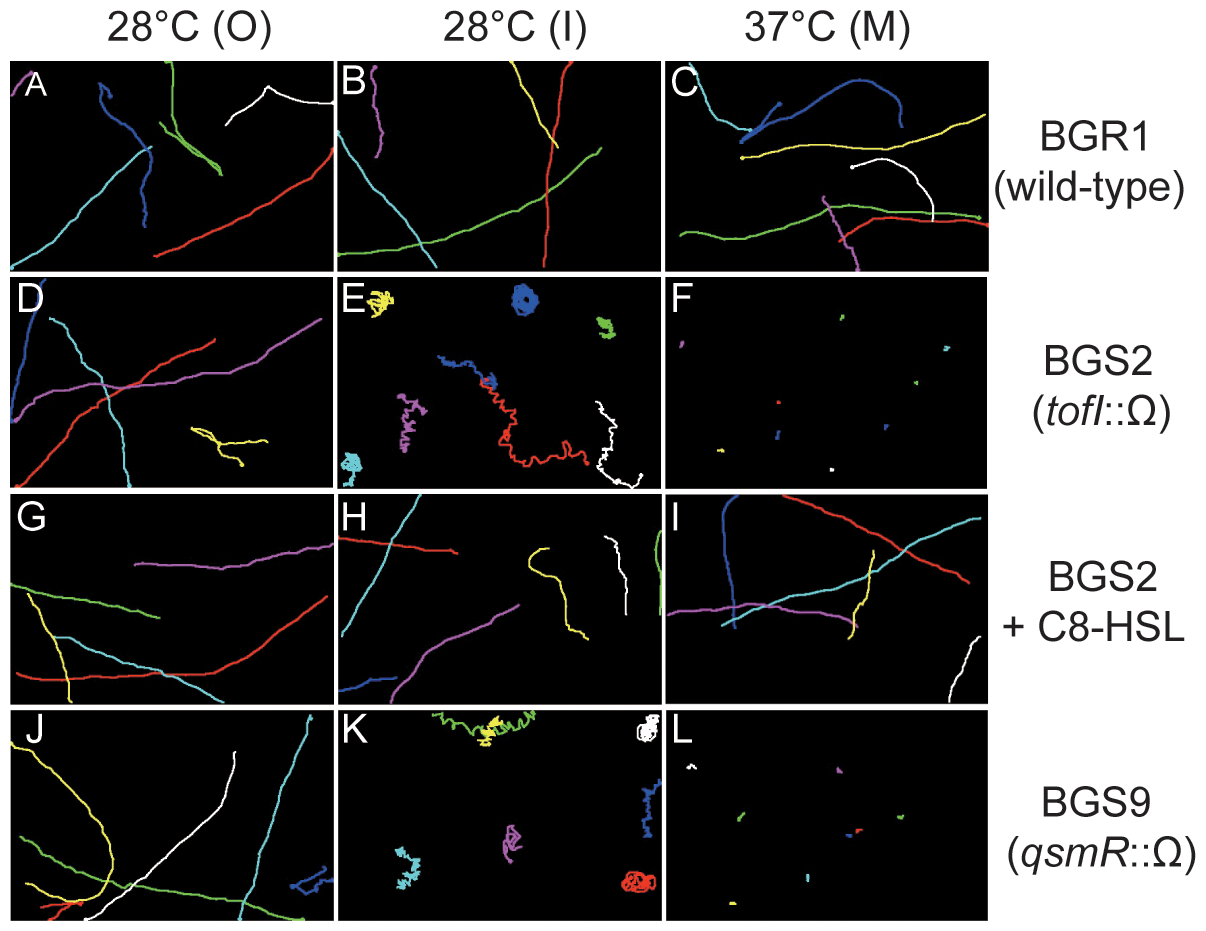
Tracking the movement of individual cells of the wild-type BGR1 and the QS mutants. Cell samples were taken from the O, I, and M regions of 0.3% soft agar plates for 20 h at 28°C and 37°C. Individual cell movement of the wild-type BGR1, the *tofI* mutant BGS2, and the *tofI* mutant BGS2 cells supplemented with 1 µM of C8-HSL, and the *qsmR* mutant BGS9 cells was determined by the manual tracking plug-in of ImageJ program. Each colored line shows the path of a different cell over a 2-s period. Swimming movement of individual BGR1 cells (A–C) Swimming movement of individual BGS2 cells (D–F). Swimming movement of individual BGS2 cells supplemented with 1 µM of C8-HSL (G–I). Swimming movement of individual BGS9 mutant cells (J–L).

To determine whether the abnormal flagellar morphology observed in the TEM images of the QS-defective mutants affects cell swimming motility, we observed individual cell movement of the strains in LB broth at 28°C and 37°C under a phase-contrast microscope. Using ImageJ, we traced individual cell movement for 2 s (5–9 examples of representative cell movement are shown in Figure 5). BGR1 wild-type cells showed typical directional swimming movement, regardless of temperature or location within the swim ring (Figures 5A–C; Movies S1, S2, S3). In the O region of the swim ring, the two QS mutants exhibited swimming motility similar to that of the wild-type BGR1 at 28°C (Figures 5D and J; Movies S4 and S10). In contrast, QS mutant cells sampled from the I region exhibited tumbling and spinning movements at 28°C (Figures 5E and K; Movies S5 and S11). The exogenous addition of 1 µM C8-HSL to the soft agar assay plates led to the recovery of normal swimming motility, similar to that of the wild-type BGR1, at both 28°C and 37°C (Figures 5G–I; Movies S7, S8, S9). The abnormal swimming motility of the QS mutants was correlated with the formation of lateral flagella at 28°C. QS mutant cells grown at 37°C were nonmotile, as expected, given that they were aflagellate (Figures 5F and L; Movies S6 and S12).

### FlhF is necessary for directional swimming movement

**Figure 6:**
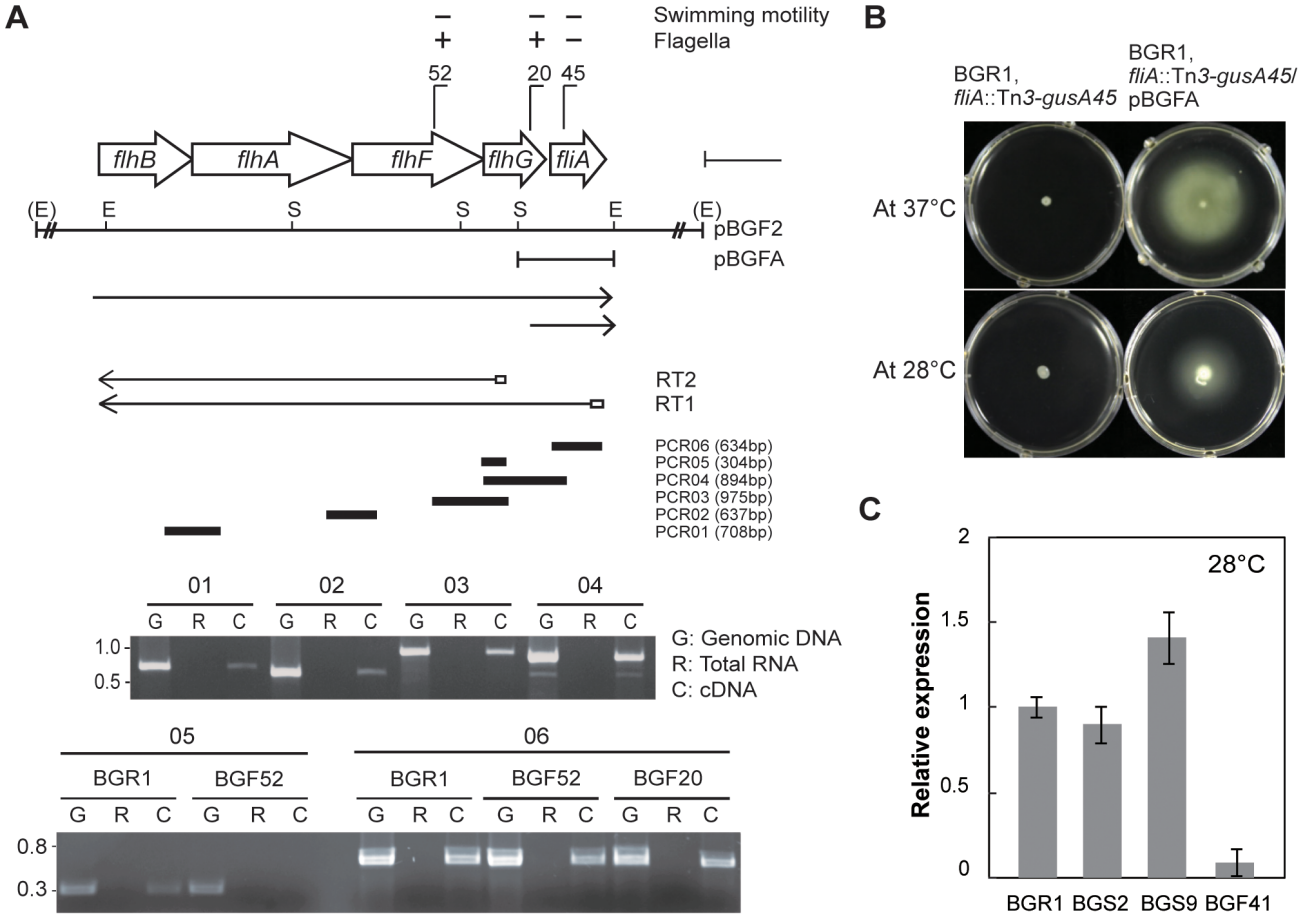
Genetic organization of *flhBAFG* and *fliA* genes, and *flhF* gene expression at 28°C. **A**. Genetic organization and transcriptional units of *flhBAFG* and *fliA*, with arrows showing the direction of transcription. Recognition sites for *Eco*RI and *Stu*I are indicated by E and S, respectively. Vertical bars on the map denote the positions and orientation of the Tn*3*-*gusA* insertion. The two lines with arrowheads beneath the restriction enzyme map indicate the direction and extent of transcription. cDNA synthesized by reverse transcriptase with primers RT1 and RT2 was analyzed by PCR. Thick bars indicate the six expected PCR products, which were verified by electrophoresis on 1.5% agarose gel. **B**. Photographs of swim assay plates showing the swimming motility of the BGR1 mutant *fliA*::Tn*3*-*gusA45* and complementation with pBGFA carrying the *fliA* gene at 28°C and 37°C. **C**. Expression levels of the *flhF* gene in the wild-type BGR1, the *tofI* mutant BGS2, and the *qsmR* mutant BGS9, and the *flhC* mutant BGF41 at 28°C, based on qRT-PCR analysis. Vertical lines indicate the standard deviations of three independent experiments.

Because FlhF has been reported as a SRP-type GTPase that is critical for flagellar formation in other bacteria, we examined the functional roles of *flhF* in *B*. *glumae*. The *flhBAFG* and *fliA* genes were co-transcribed as confirmed via RT-PCR analysis (PCR01, PCR02, PCR03, and PCR04 reactions); however, Tn*3*-*gusA* insertions in the *flhF* gene did not affect *fliA* expression (Figure 6A). This result indicates that Tn*3*-*gusA* insertion in the *flhF* gene either does not have a polar effect on downstream *fliA* gene expression, or that the *fliA* gene has its own promoter. To test these two possibilities, cDNA was synthesized from mRNA of BGR1 *flhF*::Tn*3*-*gusA52* (BGF52) by reverse transcriptase with a primer RT1. RT-PCR analysis (PCR05 reaction) confirmed that Tn*3*-*gusA* insertion in the *flhF* gene has a polar effect on downstream gene expression (Figure 6A). However, when the same cDNA was used to amplify the *fliA* gene in the PCR06 reaction, positive amplification was detected (Figure 6A). Furthermore, when pBGFA (which carries the full length of the *fliA* gene, with the upstream region potentially carrying its own promoter) was transferred to the BGR1 *fliA*::Tn*3*-*gusA45*, the ability of the cells to swim was restored to the mutant, implying the formation of flagella (Figure 6B). These results indicate that the *fliA* gene might have its own promoter.

**Figure 7:**
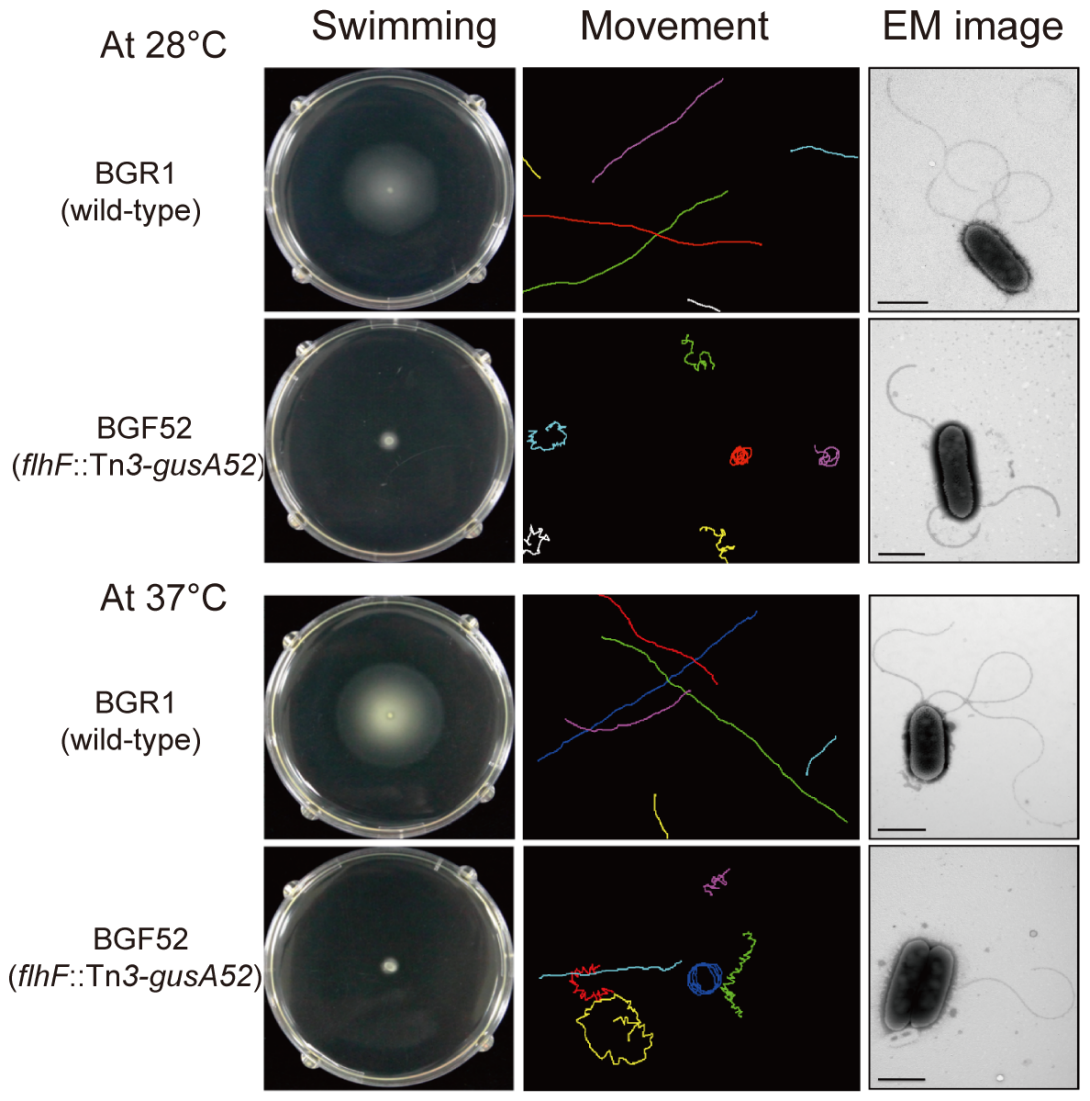
Swimming motility, movement, and flagellar morphology of the *flhF* mutant. After culturing on swim assay plates for 20-in of the ImageJ program. Each colored line shows the path of a different individual cell over a 2-s period. Flagellar morphologies of the wild-type BGR1 and the *flhF* mutant were determined by analyzing TEM images. Bars = 1 µm.

To determine whether expression of *flhF* is still under the control of FlhDC at 28°C, we measured the expression levels of the *flhF* gene in the wild-type BGR1, the BGS2 mutant, and the BGS9 mutant. The *flhF* gene was expressed in the two QS mutants in a FlhDC-dependent manner at 28°C (Figure 6C). This result was consistent with the fact that the *flhC* gene is expressed in the QS mutants at 28°C. The *flhF* mutant of the wild-type BGR1 showed no swimming motility in the swim assay plates at 28°C or 37°C; however, it possessed lateral flagella displaying spinning and nonlinear movements, as observed by phase-contrast microscopy (Figure 7).

### Localization of FlhF in QS mutants

**Figure 8:**
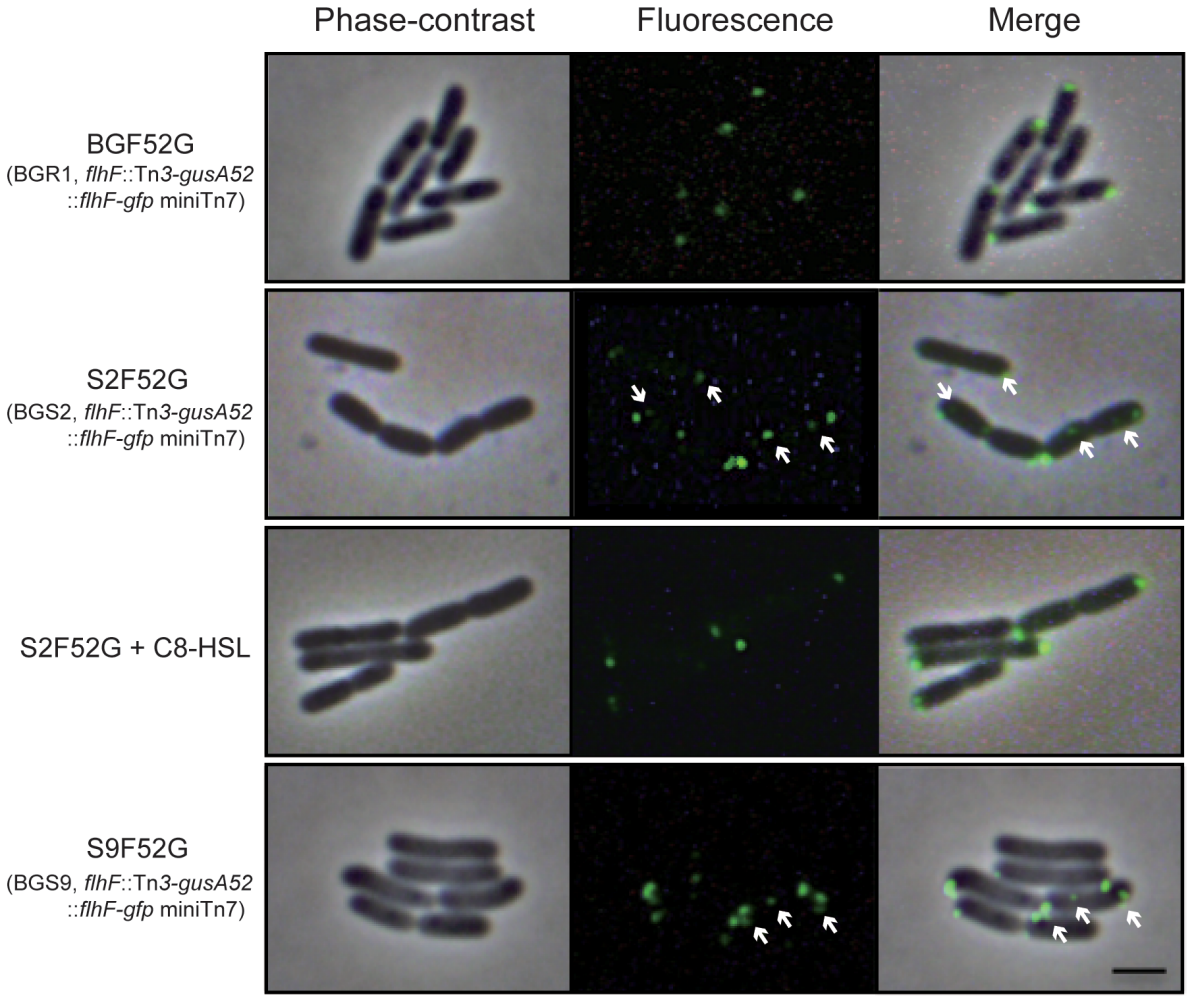
Localization of the FlhF-eGFP in wild-type BGR1 and the QS mutant backgrounds. Phase-contrast and fluorescence images of the FlhF-eGFP protein were visualized using an Olympus BX53 phase-contrast microscope and the same microscope equipped with a GFP filter, respectively. White arrows indicate non-polarly localized FlhF-eGFP proteins. Bar = 2 µm.

To determine the localization of FlhF in the QS mutants, a single copy of the *flhF*-*egfp* fusion gene was specifically integrated between 25 and 26 bp, downstream of the *glmS* gene of the BGR1 *flhF*::Tn*3*-*gusA52* (BGF52), the BGS2 *flhF*::Tn*3*-*gusA52* (S2F52), and the BGS9 *flhF*::Tn*3*-*gusA52* (S9F52) mutants. We confirmed that integration of a single copy of the *flhF*-*egfp* fusion gene complemented phenotypes of BGF52G, S2F52G, and S9F52G. We examined FlhF-eGFP protein expression in the presence (BGF52) and absence of the QS system (S2F52 and S9F52) at 28°C using fluorescence microscopy. In BGF52G cells, the FlhF-eGFP protein was localized at the cell pole (Figure 8). In comparison, in S2F52G and S9F52G cells, the FlhF-eGFP protein was observed at the cell pole and at locations around the entire cell surface (Figure 8). The exogenous addition of 1 µM C8-HSL to S2F52G mutant cells recovered the normal polar localization of FlhF (Figure 8).

## Discussion

Rice panicle blight occurs when rice flowers are infected with *B*. *glumae*, and the disease is becoming prevalent when temperature is exceeding 30°C [34], [35]. Therefore, temperature and cell motility are important factors for the successful infection of rice flowers by *B. glumae* [24]. In our previous report, we showed that non-motile *fliA* and *flhD* mutants are less virulent [24]. Yet, paddy fields are exposed to major temperature fluctuations during the rice growing period; hence, *B*. *glumae* is frequently exposed to temperatures below 37°C, which is the optimum growth temperature for this species *in vitro*. In addition, *B*. *glumae* does not produce its major virulence factor, phytotoxin toxoflavin, at temperatures below 30°C [36]. Therefore, it is important to study the motility characteristics of *B*. *glumae* at temperatures below 30°C. In the present study, we observed that the wild-type BGR1 produced noticeably more polar flagella at 28°C compared to 37°C. This result indicates that 28°C, rather than 37°C, might be the ideal temperature for successful movement and infection by *B*. *glumae*, which is a major contrast to previously documented laboratory conditions. This finding also implies that the two known virulence factors of *B*. *glumae*, toxoflavin and motility, might act at different temperatures to successfully infect rice.

In this study, we hypothesized that genetic changes in bacteria might produce a heterogeneous population of cells possessing polar and nonpolar flagella; hence, we documented the flagellar morphology of *B*. *glumae* in different swimming areas of the agar plate. Traditional bacterial swimming assays are often characterized by the diameter of the swim ring on soft agar plates. This type of assay allows the interpretation of bacterial growth and swimming motility at massive bacterial population levels after a certain period of incubation. In bacterial swimming motility assays, the growth rate and age of cells, availability of oxygen, physical barriers (e.g., polysaccharides produced as bacterial byproducts), and other stress conditions may affect bacterial formation on the swim ring [37], [38]. Relatively young and actively motile cells generally move forward and reside at the edge of the swim ring; however, this type of swimming motility assay does not accurately reveal the swimming ability of individual cells in a bacterial population occupying a liquid environment. Therefore, we examined the flagellar morphology of individual cells collected from the O, M, and I regions of the swim ring.

We recorded the presence of a heterogeneous bacterial population for QS mutants, which had both polar and nonpolar flagella. Previously, we had reported that QS mutants are able to swim and form polar flagella at 28°C [24], which is quite different from the observations found in this study. In our previous study, we sampled QS mutant cells from the edge of the swimming ring. In contrast, in this study, we observed flagellar morphology at the O, M, and I regions, based on swimming distance. These results demonstrate that it is important to investigate flagellar morphology at different regions of the swimming ring, particularly when expecting a population to contain individual cells with different types of flagellar morphology.

We found that QS-dependent flagellar formation in *B*. *glumae* changed under different temperature regimes. Several previous studies have reported the involvement of QS in flagellar biogenesis; however, it was not known whether QS is involved in generating the morphological changes of flagella, or whether QS interacts with environmental factors to determine flagellar morphology. QS, in addition to the various environmental factors and regulators involved in flagellum biosynthesis, has both positive and negative effects on the regulation of flagellar biogenesis and motility in bacteria [39]. In *Yersinia enterocolitica* and *B. glumae*, the positive regulation of flagellar gene expression is achieved by the QS-dependent activation of *flhDC* [21], [24]. In *Sinorhizobium meliloti* and other bacteria, such as *Erwinia chrysanthemi* pv. *zeae*, QS downregulates the expression of flagellar genes, causing QS-defective mutants to be hypermotile [40]. In the present study, we showed that flagellar biogenesis is independent of QS at 28°C, despite QS being required for normal polar flagellar morphogenesis of *B*. *glumae* at 28°C. These results indicate that QS and temperature operate together to modulate flagellar biosynthesis and morphology in *B*. *glumae*.

At 28°C, the degree of swimming motility on soft agar plates was very similar between the wild-type and QS mutant cells, even though the QS mutant cell populations contained mixtures of polarly and nonpolarly flagellated cells. This finding indicates that when polarly flagellated bacteria fail to respond to the high density of the community, no flagella formed at 37°C or unusual nonpolarly flagella are formed at 28°C. When considering the social aspects of these phenotypes, QS might serve as a means of dispersal for individual cells in the group when bacterial cell densities are high. However, because QS mutants mimic low cell density, this social response is hindered by aflagellated or abnormal flagellated cells at 28°C. This phenomenon presents another example of the importance of QS in regulating bacterial social behavior. However, we cannot completely rule out a possibility that different metabolic status in different locations in the swimming assay plates might affect flagellar morphogenesis.

FlhF is involved in both flagellar biosynthesis and flagellar arrangement in polarly flagellated bacteria. In *V. cholerae* and *Campylobacter jejuni*, the *flhF* deletion mutant does not have flagella, and so does not exhibit swimming motility [11], [41]. In contrast, the *flhF* deletion mutant of *P. aeruginosa* exhibits a random arrangement of flagella and a loss of motility [14]. Moreover, the overexpression of FlhF causes hyperflagellation in *P. putida* [13]. These observations indicate that optimal levels of *flhF* expression and the localization of FlhF are critical for normal flagellar formation. This study showed that FlhF is not essential for flagellar biosynthesis, but that it plays an important role in determining *B*. *glumae* flagellar morphology. This led us to hypothesize that a deficiency of QS causes the mislocalization of proteins involved in flagellar formation, such as FlhF in *B*. *glumae*. In fact, FlhF was mislocalized in QS mutants. However, it is not clear whether the mislocalization of FlhF is a direct cause of abnormal flagellar formation, or whether the loss of FlhF function causes the mislocalization of other flagellar basal body proteins. Because normal polar flagella formation is important for the virulence of *B*. *glumae* [24], abnormal flagellar morphology might result in less efficient swimming movement by *B*. *glumae* during the invasion of rice flowers, thus hindering invasion success. This characteristic could be used to develop biological management techniques to enhance crop production and reduce the spread of economically damaging rice panicle blight.

## Materials and Methods

### Bacterial strains, plasmids, and growth conditions

The strains and plasmids used in this study are listed in Table S1. The cells of *B. glumae* and *E. coli* strains were grown at 37°C and 28°C in Luria Bertani (LB) broth. The broth contained tryptone (10 g/l), yeast extract (10 g/l), and NaCl (5 g/l), in the presence and absence of 1.5% (wt/vol) agar. Antibiotics were used at the following concentrations: 100 µg/ml rifampicin, 50 µg/ml kanamycin, 100 µg/ml spectinomycin, 10 µg/ml tetracycline, and 50 µg/ml ampicillin.

### Motility assay

The swim assay was performed at 28°C and 37°C in plates containing LB broth with 0.3% Bacto™ agar. Each strain was inoculated in 2 ml of LB broth, and incubated at 37°C for 14 h, with shaking. The cultures were subcultured and incubated for 6 h at 37°C, with shaking. The cells were harvested, washed twice with fresh LB broth, and suspended in 100 µl of LB broth. An 1-µl volume of each cell suspension was dipped in a swim assay plate and incubated for 20 h. To observe the number and placement of flagella, cells of *B. glumae* were collected from three regions of the agar plate; specifically, the outside (O; edge of swimming ring), inside (I; center of swimming ring), and middle (M; middle of O and I). These regions were divided based on the swim distance of bacteria on the soft agar plate (Figure 1A).

### Transmission electron microscopy (TEM)

Cells of *B. glumae* were cultured on swim assay plates for 20 h at 28°C and 37°C. A Formvar/carbon-coated grid was placed onto the O, M, and I regions of the swimming ring on the assay plate. The cells were negatively stained with a 2% aqueous solution of phosphotungstic acid (pH 7.4). After 1 min, the liquid was removed with filter paper, and the grid was washed twice with distilled water. The cells were examined by TEM (JEM 1010; Jeol).

### Cell movement records

To observe cell movement, cells were sampled from the O, M, and I regions of the motility ring on the assay plate, and observed under a phase-contrast microscope (BX53; Olympus). Video clips were obtained by a 1.5 M-pixel CCD digital camera (FOculus). Each video clip ran for a total of 30 s using a phase-contrast microscope (Olympus BX35) with a 40× phase-contrast objective to observe a large field of view. QuickTime video recordings were analyzed by the ImageJ program (National Institutes of Health) using the manual tracking plug-in.

### β-Glucuronidase (GUS) assay

The β-glucuronidase gene was used as a reporter gene to measure gene expression. All *B. glumae* BGR1 derivatives were grown in 2 ml of LB broth for 12 h at 37°C and for 16 h at 28°C, with shaking. Cells were collected by centrifugation, suspended in 0.5 ml of GUS extraction buffer, and lysed by sonication (VCX-400 sonicator; Sonics and Materials). β-Glucuronidase enzyme activity was assayed as previously described [32].

### Marker-exchange mutagenesis

The pBGF2 plasmid was mutagenized with Tn*3*-*gusA*, followed by marker-exchange into the wild-type BGR1, the *tofI*::Ω mutant BGS2, and the *qsmR*::Ω mutant BGS9, as described previously [24]. The marker exchange was confirmed by Southern hybridization analysis.

### Quantitative real-time polymerase chain reaction (qRT-PCR)

An RNeasy Mini Kit (Qiagen) was used to isolate total RNA from *B*. *glumae* cells in LB broth cultured for 10 h at 37°C and for 14 h at 28°C. Isolated RNA was treated with DNase I (Ambion) for 30 min at 37°C. Total RNA (0.5 µg) was reverse transcribed to cDNA using Superscript III reverse transcriptase at 55°C for 1 h, followed by 15 min at 75°C, according to the manufacturer's instructions (Invitrogen). For RT-PCR analysis, primers of RT1, RT2, PCR01F, PCR01R, PCR2F, PCR02R, PCR03F, PCR03R, PCR04F, PCR04R, PCR05F, PCR05R, PCR06F, and PCR06R were used (Table S2). For qRT-PCR analysis, the primers of flhC-cDNA, flhF-cDNA, flhC-qRTF, flhC-qRTR, flhF-qRTF, and flhF-qRTR were used (Table S2). qRT-PCR analysis was performed according to the manufacturer's instructions except using SsoFast™ EvaGreen Supermix (Bio-Rad) with 25 ng of cDNA as template. qRT-PCR was performed using a thermal cycler (Model C1000™; Bio-Rad) under the following conditions: 98°C for 2 min, followed by 35 cycles at 98°C for 20 s, 65°C for 30 s, and 72°C for 30 s. The 16S rRNA gene was used for data normalization.

### Construction of *flhF*–*egfp* gene fusion

To study the localization of FlhF in *B. glumae*, a translational fusion of FlhF to EGFP was placed under the control of the *flhB* promoter. The 359-bp promoter region upstream of *flhB* was PCR-amplified, using pBGF2 as the template DNA and the oligonucleotide primers PFlhBF1 and PFlhBR1 (Table S2). This procedure introduced unique *Kpn*I and *Nde*I sites to the end of the PCR product, which was then cloned into the *Sma*I site of pBluescript II SK(+) to generate pPFlhB (Table S1).

The coding region of *flhF* that lacked the stop codon was PCR-amplified using pBGF2 as the template DNA and the oligonucleotide primers FlhFF1 and FlhFR1 (Table S2), which introduced unique *Nde*I and *Xho*I sites to the end of the PCR product, which was then cloned into the *Sma*I site of pBluescript II SK(+) to generate pFlhF (Table S1). The inserted DNA was sequenced with the T7 promoter primer to confirm that the PCR products did not contain errors.

The 1721-bp *Nde*I–*Xho*I fragment of pFlhF was subcloned into the same sites of pPFlhB, to produce pPflhF-S (Table S1). The *flhF* gene that contained the *flhB* promoter region was excised from pPflhF-S by *Xho*I and *Sac*I, and cloned into the same sites of pJW23 containing the *egfp* gene, to produce pPFG1 (Table S1). A fragment containing the *flhF–egfp* fusion gene was excised by *Sac*I and *Eco*RI, and subcloned into pUC18R6KT miniTn*7*T-Tc, to generate the final construct, pPFG2 (Table S1). pPFG2 was integrated into the genomes of BGF52, S2F52, and S9F52 (Table S1).

### Integration of *flhF*::*egfp* into the *B. glumae* genome

Mini-Tn*7* carrying the *flhF–egfp* fusion gene was integrated into the *glmS* gene (which encodes glucosamine 6-phosphate synthetase) of *B. glumae* by four-parental mating conjugation, as previously described for *B*. *mallei* ATCC 23344 [33]. Cells of *B. glumae*, *E. coli* DH5α λ*pir*(pTNS2), *E. coli* HB101(pRK2013), and *E. coli* S17-1 λ*pir*(pPFG2) were cultured in 2 ml of LB broth at 37°C for 14 h, with shaking. The cultures were subcultured and incubated for 2 h at 37°C, with shaking, and were subsequently washed with LB broth. Prepared cells were mixed and incubated on LB agar plates containing 20 mM MgSO_4_ for 14 h. These cells were suspended in 0.5 ml of LB broth and incubated on LB agar plates containing 5 µg/ml tetracycline. Proper integration was verified by PCR using the primer pairs glmS-down/P_Tn7R_ and glmS-down/1g32800-up (Table S2).

### Imaging of FlhF-eGFP fusion protein in live cells

The cells were incubated at 28°C on 0.3% soft agar plates, and sampled from the edge of the motility ring. FlhF-eGFP protein in cells was visualized using an Olympus BX53 phase-contrast microscope equipped with a GFP filter. Images were captured using the Focus Lite program (Focus).

## Supporting Information

Movie S1 **Swimming movements of BGR1 sampled from the O region at 28°C.**
(AVI)

Movie S2 **Swimming movements of BGR1 sampled from the I region at 28°C.**
(AVI)

Movie S3 **Swimming movements of BGR1 at 37°C.**
(AVI)

Movie S4 **Swimming movements of BGS2 sampled from the O region at 28°C.**
(AVI)

Movie S5 **Swimming movements of BGS2 sampled from the I region at 28°C.**
(AVI)

Movie S6 **Swimming movements of BGS2 at 37°C.**
(AVI)

Movie S7 **Swimming movements of BGS2 (supplemented with C8-HSL) sampled from the O region at 28°C.**
(AVI)

Movie S8 **Swimming movements of BGS2 (supplemented with C8-HSL) sampled from the I region at 28°C.**
(AVI)

Movie S9 **Swimming movements of BGS2 (supplemented with C8-HSL) at 37°C.**
(AVI)

Movie S10 **Swimming movements of BGS9 sampled from the O region at 28°C.**
(AVI)

Movie S11 **Swimming movements of BGS9 sampled from the I region at 28°C.**
(AVI)

Movie S12 **Swimming movements of BGS9 at 37°C.**
(AVI)

Table S1 **Bacterial strains and plasmids.**
(DOCX)

Table S2 **Primers used in this study.**
(DOCX)

References S1 **Supporting information references.**
(DOCX)
