# Supplementary material for: Quorum Sensing Controls Flagellar Morphogenesis in Burkholderia glumae
Source: PLoS One. 2014 Jan 8;9(1):e84831. doi: 10.1371/journal.pone.0084831 (PMC3885665; doi:10.1371/journal.pone.0084831)
Supplement: Table S1 — Bacterial strains and plasmids. (DOCX) [file pone.0084831.s013.docx]

**Table S1 Bacterial strains and plasmids**

| Strain or plasmid | Description^a^ | Source or reference |
| --- | --- | --- |
| ***Escherichia coli*** |  |  |
| DH5α | F^−^ Φ80d*lac*ZΔM15 Δ(*lacZYA-argF*)*U169* *endA1 recA1* *hsd1 hsdR17*(r_K_^−^ m_K_^+^) *deoR thi-1 supE44* λ^−^ *gyrA96 relA1* | Gibco-BRL |
| DH5α λ*pir* | F^−^ Φ80d*lac*ZΔM15 Δ(*lacZYA-argF*)*U169* *endA1 recA1* *hsd1 hsdR17*(r_K_^−^ m_K_^+^) *deoR thi-1 supE44* λ^−^ *gyrA96 relA1*, *λpir* | [1] |
| HB101 | F^−^ *mcrB mrr hsdS20*(r_B_^−^ m_B_^−^) *recA13 leuB6 ara-14 proA2 lacY1 galK2 xyl-5 mtl-1 rpsL20* (Sm^r^) *supE44* λ^−^ | Gibco-BRL |
| S17-1 λ*pir* | Tp^r^ Sm^r^ *recA*, *thi*, *pro*, *hsdR*^-^*M*^+^ RP4::2-Tc::Mu:Km ::Tn*7*, *λpir* | [1] |
| ***Burkholderia glumae*** |  |  |
| BGR1 | Wild-type, Rif^R^ | [2] |
| BGS2 | BGR1 *tofI*::Ω | [3] |
| (Continued)  BGS9 | BGR1 *qsmR*::Ω | [4] |
| BGF41  BGF49/S2F49/ S9F49 | BGR1 *flhC*::Tn*3*-*gusA41*  BGR1 *fliC*::Tn*3*-*gusA49/*BGS2 *fliC*::Tn*3*-*gusA49/*BGS9 *fliC*::Tn3-*gusA49* | [4]  [4] |
| BGF28/S2F28/S9F28 | BGR1 *flgK*::Tn*3*-*gusA28/*BGS2 *flgK*::Tn*3*-*gusA28/*BGS9 *flgK*::Tn3-*gusA28* | [4] |
| BGF52/S2F52/S9F52 | BGR1 *flhF*::Tn3-*gusA52/* BGS2 *flhF*::Tn3-*gusA52/*BGS9 *flhF*::Tn3-*gusA52* | This study |
| BGF20 | BGR1 *flhG*::Tn3-*gusA20* | [4] |
| BGF52G/S2F52G/S9F52G | BGF52::pFlhF-eGFP-miniTn*7*/BGF52::pFlhF-eGFP-miniTn*7*/BGF52::pFlhF-eGFP-miniTn*7* | This study |
| BGF45 | BGR1 *fliA*::Tn*3*-*gusA45* | [4] |
| **Plasmids** |  |  |
| pBluescript II SK(+) | Cloning vector: phagemid, pUC derivative, Amp^R^ | Stratagene |
| pGEM-T Easy | PCR cloning vector, Amp^R^ | Promega |
| pRK2013 | Tra^+^, ColE1 replicon, Km^R^ | [5] |
| pBGF2 | 23-kb DNA fragment harbouring *flh* and *che* in pLAFR3 | [4] |
| pBGFA | 1.2-kb DNA fragment harbouring *fliA* and its promoter region cloned into pLAFR6 | This study |
| pUC18R6K-miniTn*7*T-Tc | Mobilizable mini-Tn*7*-Tc vector, Amp^R^, Tet^R^ | [1] |
| pTNS2 | Plasmid expressing *tnsABCD* from P*_lac_,* Amp^R^ | [6] |
| pJW23 | Coding region of *egfp* was cloned into pGEM-T Easy vector | This study |
| pPFlhB | 356-bp PCR product containing the promoter region of *flhH* inserted into the *Sma*I site of pBluscript II SK(+) | This study |
| pFlhF | 1721-bp PCR product of the coding region of *flhH* inserted into the *Sma*I site of pBluscript II SK(+) | This study |
| pPflhF-S | 1.7-kb *Nde*I–*Xho*I DNA fragment harbouring *flhF* cloned into pPFlhB | This study |
| pPFG1 | 2.1-kb *Sac*I–*Xho*I DNA fragment harbouring P_flhB_*flhF* cloned into pJW23 | This study |
| pPFG2 | 2.8-kb *Sac*I–*Eco*RI DNA fragment harbouring P_flhB_*flhF–egfp* cloned into pUC18R6K-miniTn*7*T-Tc |  |

^a^ Amp^R^, ampicillin resistance; Km^R^, kanamycin resistance; Rif^R^, rifampin resistance;

Sp^R^, spectinomycin resistance; Tet^R^, tetracycline resistance
