# Supplementary material for: Quorum Sensing Controls Flagellar Morphogenesis in Burkholderia glumae
Source: PLoS One. 2014 Jan 8;9(1):e84831. doi: 10.1371/journal.pone.0084831 (PMC3885665; doi:10.1371/journal.pone.0084831)
Supplement: Table S2 — Primers used in this study. (DOCX) [file pone.0084831.s014.docx]

**Table S2. Primers used in this study**

| **Primer name** | **Sequence (5′→3′)^a^** |
| --- | --- |
| flhC-cDNA | CACAGCCCGCAGACGAAAC |
| flhF-cDNA | GGATCCGGCCGAAGATGC |
| RT1 | GCTGTGGAGCTGGCACAC |
| RT2 | CAAGGCGTTCATCGTCG |
| 16S rRNAF | TCTGAGAGGACGACCAGCCA |
| 16S rRNAR | CGAAGGCCTTCTTCACACAC |
| flhC-qRTF | CTTCGACTCGGGGATGCTG |
| flhC-qRTR | CACAGCCCGCAGACGAAAC |
| flhF-qRTF | CGTGGGCAAGACCAC |
| flhF-qRTR | GGATCCGGCCGAAGATGC |
| PCR01F | CAAGGGCGTGAACCTG |
| PCR01R | CAGGAAGTGGCCGAAC |
| PCR02F | CGATCACCCAGCAGTGG |
| PCR02R | CACCGACGAGAACACGTC |
| PCR03F | GGTGGTGCAGGCCTATC |
| PCR03R | CAAGGCGTTCATCGTCG |
| PCR04F | GCGAACTCGTGCACACC |
| PCR04R | GCTGTGGAGCTGGCACAC |
| PCR05F | CGAATCACCGATCAGGC |
| PCR05R | CAAGGCGTTCATCGTCG |
| PCR06F | GGTGCTCGCGAAATATGCGC |
| PCR06R | GCTGTGGAGCTGGCACAC |
| PFlhBF1 | AACGGTACCTAGGACGGCCAGCGGAGCC |
| PFlhBR1 | CAGCATATGTGCAGCCTCCGGTCGGAGACG |
| FlhFF1 | CAGCATATGAACATCCGCAAATTCACCGGCCC |
| FlhFR1 | ACCCTCGAGTCCAAAGTAGACCTCGTGATGTCCGG |
| glmS-down | AGCCGCAGATCATCGCCTG |
| 1g32800-up | CCACGCATCGAAATCCTC |
| P_Tn7R_ | CACAGCATAACTGGACTGATTTC |

^a^ Restriction sites are underlined.
