# Supplementary material for: Quorum Sensing Controls Flagellar Morphogenesis in Burkholderia glumae
Source: PLoS One. 2014 Jan 8;9(1):e84831. doi: 10.1371/journal.pone.0084831 (PMC3885665; doi:10.1371/journal.pone.0084831)
Supplement: References S1 — Supporting information references. (DOCX) [file pone.0084831.s015.docx]

**References**

1. Choi KH, DeShazer D, Schweizer HP (2006) Mini-Tn*7* insertion in bacteria with multiple *glmS*-linked attTn*7* sites: example *Burkholderia mallei* ATCC 23344. Nat Protocols 1:162–169.

2. Jeong Y, Kim J, Kim S, Kang Y, Nagamatsu T, et al. (2003) Toxoflavin produced by *Burkholderia glumae* causing rice grain rot is responsible for inducing bacterial wilt in many field crops. Plant Dis 87:890–895.

3. Kim J, Kim JG, Kang Y, Jang JY, Jog GJ, et al. (2004) Quorum sensing and the LysR-type transcriptional activator ToxR regulate toxoflavin biosynthesis and transport in *Burkholderia glumae*. Mol Microbiol 54:921–934.

4. Kim J, Kang Y, Choi O, Jeong Y, Jeong JE, et al. (2007) Regulation of polar flagellum genes is mediated by quorum sensing and FlhDC in *Burkholderia glumae*. Mol Microbiol 64:165–179.

5. Keen NT, Tamaki S, Kobayashi D, Trollinger D. 1988. Improved broad-host-range plasmid for DNA cloning in gram negative bacteria. Gene 70:191–197.

6. Kim J, Heindl JE, Fuqua C (2013) Coordination of division and development influences complex multicellular behavior in *Agrobacterium tumefaciens*. PLoS One 8: e56682.
